# Timosaponin AIII induces antiplatelet and antithrombotic activity via Gq-mediated signaling by the thromboxane A2 receptor

**DOI:** 10.1038/srep38757

**Published:** 2016-12-09

**Authors:** Yue Cong, Limei Wang, Renjun Peng, Yang Zhao, Fan Bai, Chao Yang, Xiaolan Liu, Daqian Wang, Baiping Ma, Yuwen Cong

**Affiliations:** 1 Department of Pathophysiology, Beijing Key Laboratory for Radiobiology (BKLRB), Beijing Institute of Radiation Medicine, No. 27 Taiping Road, Beijing 100850, China; 2 National Resource Center for Chinese Materia Medica, Institute of Clinical Pharmacy of Beijing Municipal Health Bureau, No. 13 Shuiche Alley, Beijing 100035, China; 3 Department of Biotechnology, Beijing Institute of Radiation Medicine, No. 27 Taiping Road, Beijing 100850, China

## Abstract

The thromboxane (Tx) A_2_ pathway is a major contributor to the amplification of initial platelet activation and is therefore a key drug target. To identify potent small-molecule inhibitors of the thromboxane prostaglandin (TP) receptor, we screened a small steroidal saponin library using U46619-induced rat platelet aggregation assays. Timosaponin AIII (TAIII) was identified as a potent inhibitor of U46619-induced rat platelet aggregation and exhibited superior selectivity for the TP receptor versus other G protein-coupled receptors and a PKC activator. TAIII inhibited U46619-induced rat platelet aggregation independent of increases in cAMP and cGMP and the inhibition of TxA2 production. Both PKC and PLC activators restored TAIII-inhibited platelet aggregation, whereas TAIII did not inhibit platelet aggregation induced by co-activation of the G_12/13_ and G_z_ pathways. Furthermore, TAIII did not affect the platelet shape change or ROCK2 phosphorylation evoked by low-dose U46619. *In vivo,* TAIII prolonged tail bleeding time, reduced the mortality of animals with acute pulmonary thromboembolism and significantly reduced venous thrombus weight. Our study suggests that TAIII, by preferentially targeting Gq-mediated PLC/PKC signaling from the TP receptor, induces stronger *in vitro* antiplatelet activity and *in vivo* antithrombotic effects and may be an excellent candidate for the treatment of thrombotic disorders.

Platelets play a key role in preventing blood loss in response to injury but are critical for the formation of pathogenic thrombi, which are responsible for the acute clinical manifestations of atherothrombotic disease. These manifestations include acute coronary syndromes, ischemic stroke, and symptomatic peripheral artery disease, which are major causes of morbidity and mortality worldwide^1^. The crucial step in both protective hemostasis and pathological thrombosis is platelet activation, which can occur via multiple pathways by the binding of specific agonists, such as thromboxane A2 (TxA2), adenosine diphosphate (ADP), and thrombin, to their corresponding receptors on the platelet surface. Current oral antiplatelet agents target the TxA2 (aspirin) and ADP (P2Y12 inhibitors such as clopidogrel, ticlopidine, and prasugrel) platelet activation pathways and significantly reduce the incidence of ischemic events in patients with atherothrombotic disease^2^. However, these agents are associated with important clinical limitations, including a high residual risk for ischemic events, elevated bleeding risk, and variable inhibition of platelet aggregation^3^. These considerations underscore the need for novel therapies that can further reduce the risk for ischemic events without exposing patients to an increased risk of bleeding.

TxA2, which is generated from membrane phospholipids via the consecutive actions of phospholipase A2 (PLA2), cyclooxygenase-1 (COX-1) and TxA2 synthase, is one of the most powerful platelet activators known^4^. TxA2 mediates its specific effect via the thromboxane prostaglandin (TP) receptor. The role of TxA2 production in arterial thrombosis has led to the clinical use of anti-TxA2 agents that either inhibit its biosynthesis and/or antagonize the TP receptor. The clinical efficacy of aspirin is based on irreversible acetylation of COX-1 and inhibition of platelet TxA2 generation^5^. Other metabolites of membrane phospholipids formed by non-enzymatic peroxidation of membrane phospholipids in platelets, cells of blood vessels and monocytes/macrophages, such as isoprostanes and hydroxyeicosatetraenoic acids (HETEs), are all TP receptor ligands^6^,^7^; thus, TP receptor antagonists have certain pharmacological advantages over aspirin in that they not only block the effect of TxA2 on platelets but also inhibit the deleterious effects of other TP ligands, such as isoprostanes and HETEs. Moreover, antagonism of TP receptors has a favorable effect on atherosclerosis progression and arterial plaque stabilization in both animals and humans^8^,^9^. Consequently, pharmaceutical interventions targeting TP receptors are important for the efficient prophylaxis/treatment of patients with a poor response to aspirin and/or clopidogrel.

Steroidal saponins are complex compounds featuring a steroid attached to a carbohydrate moiety. They are natural surfactants and detergents and possess a broad range of biological and pharmacological properties, such as hypocholesterolemic, anti-tumor, antidiabetic, anti-inflammatory and antifungal activity^10^. We have reported that pennogenin glycosides with a spirostanol structure are strong platelet agonists and that some timosaponins have strong antiplatelet activity^11^,^12^. Structure-activity relationship analysis has revealed that steroidal saponins exhibit agonistic or inhibitory effects on platelet aggregation depending on their structure. However, the effects and mechanism of steroidal saponins on platelet function, signaling, and thrombosis are unknown.

In the present study, we screened a small steroidal saponin library isolated from Chinese medicinal herbs using a turbidimetric assay based on U46619 (a TxA2 analogue)-induced rat platelet aggregation. This screen identified Timosaponin AIII (TAIII) as a selective inhibitor of the TxA2 receptor. TAIII [3-O-β-D-glucopyranosyl-(1 → 2)-β-D-galactopyranoside sarsasapogenin] is a major active steroidal saponin in *Anemarrhena asphodeloides* Bunge (Liliaceae). We determined that TAIII inhibits U46619-induced platelet aggregation by abolishing ADP secretion independent of increases in cyclic adenosine monophosphate (cAMP) and cyclic guanosine monophosphate (cGMP) and the inhibition of TxA2 production in platelets. Subsequently, we found that TAIII preferentially suppresses thromboxane receptor-mediated activation of Gq, not G_12/13_, signaling pathways. The antithrombotic activity and synergism of TAIII with known antiplatelet agents may facilitate the development of novel antithrombotic agents.

## Results

### Identification of TAIII as a Potent Inhibitor of U46619-induced Rat Platelet Aggregation

Steroidal saponins are an important class of natural products that have attracted scientific attention for their structural diversity and significant bioactivities. In our labs, more than 300 purified steroidal saponins have been isolated from Chinese medicinal herbs rich in steroidal saponins, such as *Anemarrhena asphodeloides, Paris polyphylla* Smith var. yunnanensis, *Dioscorea nipponica, Dioscorea septemloba* Thunb., *Ophiopogon japonicus*, and *Trigonella foenum-graecum*, or extracted from their biotransformation products using cyclodextrin glycosyltransferase, glucoamylase, β-glucosidase and other crude enzymes^13^. We used a turbidimetric assay based on U46619-induced rat platelet aggregation to identify potent small-molecule inhibitors of the TxA2 receptor from our steroidal saponin library. Among the evaluated steroidal saponins, TAIII isolated from the rhizomes of *Anemarrhena asphodeloides* exhibited the strongest inhibitory effect on U46619-induced platelet aggregation.

**Figure 1:**
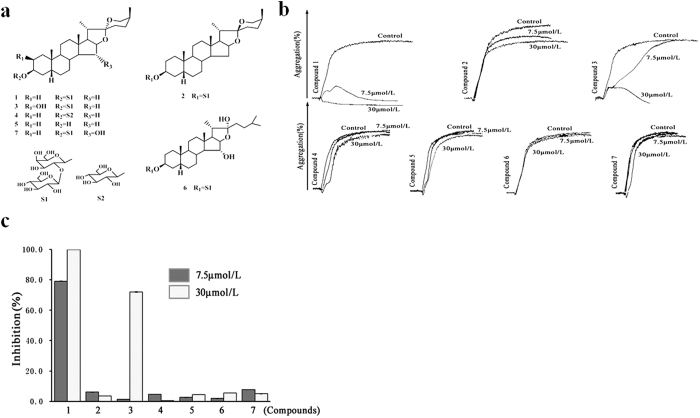
The spirostan-type 3-O-glycoside structure of Timosaponin AIII (TAIII) is an important moiety for antiplatelet aggregation activity. (**a**) Structure and key substituents of saponins included in the study. Compound 1 (TAIII), 3-O-b-D-glucopyranosyl-(1 → 2)-b-D-galactopyranoside sarsasapogenin. (**b**) Rat platelet-rich plasma (PRP) was incubated with steroidal saponins (7.5 or 30 μmol/L) or vehicle for 3 min and then stimulated with U46619 (2.5 μmol/L). Platelet aggregation was measured by the turbidimetric method. Typical real-time platelet aggregation traces are representative of three independent experiments. (**c**) Quantitative evaluation of data from at least three independent experiments. Data are presented as the percentage inhibition (mean ± SEM).

As shown in Fig. 1, the effects of TAIII analogs on U46619-induced platelet aggregation were compared. The corresponding furostanol saponin (compound 6) and steroidal sapogenin (compound 5) of TAIII (compound 1) exhibited little activity, even at the highest test concentration of 30 μmol/L. Furthermore, the introduction of a C-2 β-hydroxyl group to the aglycone of TAIII (compound 3) led to a considerable decrease in its inhibitory activity against U46619-induced platelet aggregation, whereas the introduction of a C-15 α-hydroxyl group to the aglycone of TAIII (compound 7) resulted in nearly no activity. In addition, the biotransformation metabolite compound 2, a stereoisomer of TAIII with a β-ranged CH3-21, and compound 4, produced by selective hydrolysis of β-D-glucopyranosyl from the β-D-galactopyranoside of TAIII, both exhibited little inhibitory activity. These results suggest that the spirostan-type 3-O-glycoside structure of TAIII is important for antiplatelet aggregation activity and that 15-OH and 2-OH can reduce this activity.

### TAIII is a Selective Inhibitor of TxA2-induced Platelet Activation

**Figure 2:**
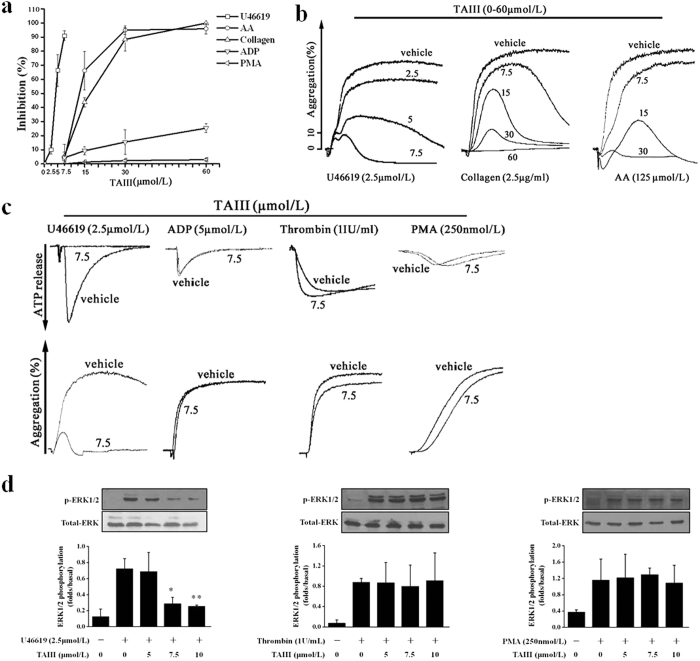
TAIII selectively inhibits TxA2-mediated platelet activation. Rat PRP was preincubated with increasing concentrations of TAIII (0–60 μmol/L) or vehicle for 3 min and then stimulated with U46619 (2.5 μmol/L), AA (125 μmol/L), collagen (1 μg/ml), ADP (5 μmol/L) or PMA (250 nmol/L). **(a)** Dose-inhibition curves for TAIII were obtained from the results of three independent experiments, and data are presented as the percentage inhibition (mean ± SEM). **(b)** Typical platelet aggregation traces are representative of three independent experiments. **(c)** Rat PRP was preincubated with 7.5 μmol/L TAIII for 3 min and then stimulated with U46619 (2.5 μmol/L), ADP (5 μmol/L), and PMA (250 nmol/L). Incubation of washed platelets with TAIII (7.5 μmol/L) was followed by stimulation with thrombin (1 IU/ml). Platelet aggregation and ATP release were measured by a turbidimetric method. Typical real-time ATP secretion and platelet aggregation traces are representative of three independent experiments. (**d)** TAIII-treated platelets were stimulated with 2.5 μmol/L U46619, 1 IU/ml thrombin or 250 nmol/L PMA to induce ERK1/2 phosphorylation. Densitometric analyses of the blots were performed using Image J software. The relative protein expression levels are presented as the ratio of phosphorylated ERK1/2 levels to the corresponding total ERK1/2 levels. Images are representative of three independent experiments, and the results are presented as the mean ± SEM. Statistical significance was determined by Student’s t-test. *p < 0.05 and **p < 0.01, compared with U46619-treated platelets.

To define the selectivity of inhibition by TAIII, we examined its ability to block platelet aggregation induced by various agonists. In rat platelet-rich plasma (PRP), U46619 (2.5 μmol/L), collagen (1 μg/ml), arachidonic acid (AA, 125 μmol/L), ADP (5 μmol/L) and phorbol 12-myristate 13-acetate (PMA, 250 nmol/L) each caused approximately 70% aggregation. As shown in Fig. 2a,b, TAIII dose-dependently inhibited U46619-, AA- and collagen-induced platelet aggregation with IC50 values of 4.36 ± 0.87 μmol/L, 16.2 ± 1.3 μmol/L and 18.71 ± 0.51 μmol/L, respectively, but had little effect on ADP- or PMA-induced aggregation, even at the maximal concentration of 60 μmol/L. Because low concentrations (1 μg/ml) of AA and collagen induced rat platelet aggregation dependent on TxA2 production^14^, we hypothesized that TAIII selectively inhibited TxA2-induced platelet aggregation. ADP released from platelets played a major role in augmenting the wave of platelet aggregation^15^. As shown in Fig. 2c, 7.5 μmol/L TAIII caused 90% inhibition of U46619-induced ATP release and platelet aggregation, suggesting that the inhibition of ATP release by TAIII parallels the inhibition of platelet aggregation. We then assessed the effect of TAIII on platelet aggregation induced by agonists that act on G protein-coupled receptors or that directly activate protein kinase C (PKC). At a concentration of 7.5 μmol/L, TAIII did not inhibit platelet aggregation or ATP release induced by ADP, thrombin or PMA. These results indicate that TAIII is a selective inhibitor of TxA2-induced platelet aggregation and ATP secretion.

Previous studies have demonstrated that acute and transient activation of ERK1/2 in platelets after stimulation with agonists such as U46619, thrombin and the PKC activator PMA plays a role in promoting platelet aggregation^16^. As shown in Fig. 2d, TAIII down-regulated U46619-stimulated ERK1/2 activation in a dose-dependent manner. At a concentration of 7.5 μmol/L, TAIII completely suppressed U46619-induced ERK1/2 phosphorylation. However, at the same concentration, thrombin- and PMA-induced activation of ERK1/2 was not affected by TAIII. These results further confirmed that TAIII is a selective inhibitor of TxA2-induced platelet activation.

### Inhibition of Platelet Aggregation by TAIII is Independent of Increases in cAMP/cGMP and the Inhibition of TxA2 Production in Platelets

**Figure 3:**
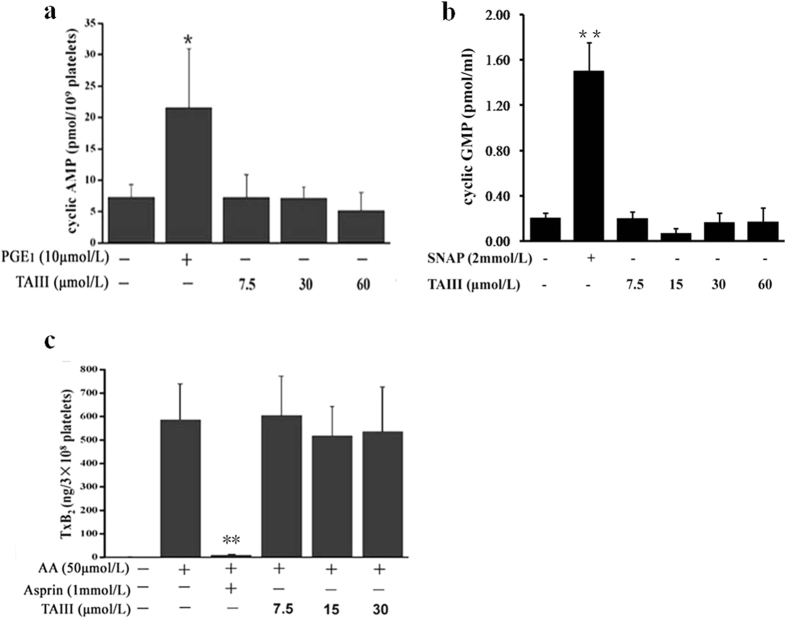
TAIII does not elevate cAMP and cGMP levels or block arachidonic acid (AA)-induced TxB2 formation in platelets. Rat PRP was incubated with PGE_1_ (10 μmol/L), SNAP (2 mmol/L) or increasing concentrations of TAIII (0–60 μmol/L) for 3 min. **(a)** cAMP and **(b)** cGMP levels were analyzed in the supernatants. The results (mean ± SEM) are from three experiments performed in triplicate. Statistical significance was determined by Student’s t-test. *p < 0.05 and **p < 0.01, compared with control platelets. **(c)** Incubation of rat PRP with aspirin (1 mmol/L) or increasing concentrations of TAIII (0–30 μmol/L) for 3 min was followed by stimulation with AA (50 μmol/L). TxB2 in the supernatant was measured by ELISA. The results (mean ± SEM) are from three experiments performed in triplicate. Statistical significance was determined by Student’s t-test. **p < 0.01, compared with the AA-treated group.

Under physiological conditions, prostacyclin (PGI_2_) and endothelium-derived nitric oxide (NO) inhibit platelet function by elevating platelet levels of cAMP and cGMP, respectively^17^,^18^. The effects of TAIII on cAMP and cGMP levels in platelets were investigated. As shown in Fig. 3a,b, TAIII did not affect the concentration of cAMP or cGMP in resting platelets, even at the highest concentration of 60 μmol/L. Upon treatment of platelets with 10 μmol/L PGE_1_ or 2 mmol/L SNAP (a NO donor), the cAMP and cGMP concentrations increased by 2.97-fold and 7.33-fold, respectively, compared to control. The effect of TAIII on TxA2 production was further investigated. As shown in Fig. 3c, AA markedly increased the TxB2 level after incubation with platelets for 6 min, and aspirin (1 mmol/L) completely inhibited AA-induced TxB2 formation. However, TAIII did not affect AA-induced TxB2 formation, even at the highest concentration of 30 μmol/L. These results indicate that TAIII inhibits rat platelet aggregation independent of increases in cAMP or cGMP and the inhibition of TxA2 production.

### TAIII Inhibits U46619-induced Platelet Aggregation by Abolishing ADP Secretion

**Figure 4:**
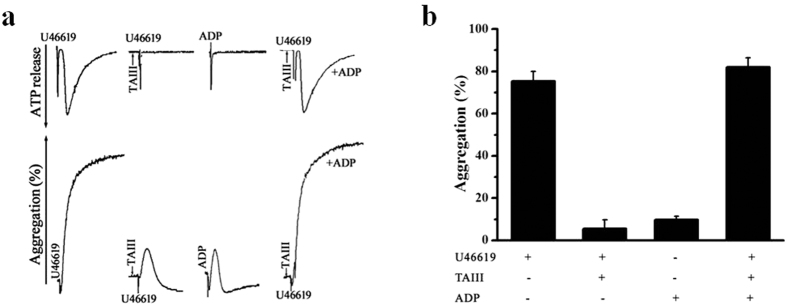
Exogenous ADP restores TAIII-inhibited platelet aggregation and dense granule secretion in response to stimulation with U46619. Rat PRP was preincubated with TAIII (7.5 μmol/L) for 3 min and then stimulated with U46619 (2.5 μmol/L) with or without immediate ADP treatment (0.25 μmol/L). (**a**) Typical real-time ATP secretion and platelet aggregation traces are representative of three independent experiments. (**b**) Quantification of aggregation (%) is presented as the mean ± SEM.

TxA2 activates the TP receptor, leading to dense granule secretion and subsequent secretion-dependent platelet aggregation through both the Gq and G_12/13_ signaling pathways. Therefore, we hypothesized that the inhibitory effects of TAIII on TxA2-induced platelet aggregation result from the inhibition of ADP secretion. To investigate this hypothesis, we examined whether restoring the low ADP concentration reverses the inhibitory effects of TAIII during U46619-induced platelet aggregation. As shown in Fig. 4, TAIII decreased secretion and platelet aggregation. The addition of a low concentration of ADP (0.25 μmol/L), insufficient to induce platelet aggregation alone, restored the aggregation induced by U46619 in TAIII-treated platelets. These results suggest that the inhibitory effects of TAIII on platelet aggregation are mainly due to the inhibition of ADP secretion from dense granules.

### TAIII Preferably Suppresses TP-mediated Activation of Gq, not G_12/13_, Signaling Pathways

**Figure 5:**
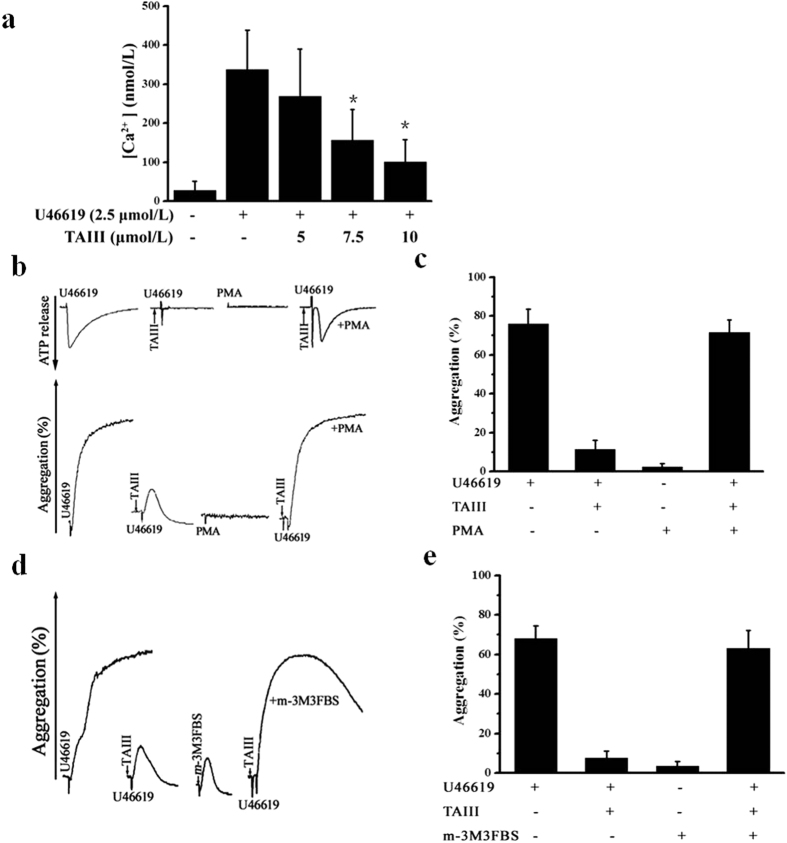
TAIII interferes with the TP-mediated Gq signaling pathway. **(a)** Platelets were preincubated with increasing concentrations of TAIII (0–10 μmol/L) for 3 min and then stimulated with 2.5 μmol/L U46619. Calcium concentration was measured by absorbance fluorometry using fura-2-AM. The results are presented as the mean ± SEM (n = 3). Rat PRP was preincubated with TAIII (7.5 μmol/L), stimulated with U46619 (2.5 μmol/L), and immediately treated with or without 20 nmol/L PMA **(b)** or 400 μmol/L *m*-3M3FBS **(d).** Typical real-time ATP secretion and platelet aggregation traces are representative of three independent experiments. Quantitative evaluation of the data from three independent experiments is shown in **(c)** and **(e)**, and the results are presented as the mean ± SEM. Statistical significance was determined by Student’s t-test. *p < 0.05, compared with U46619-treated platelets.

TxA2 stimulates the TP receptor, which activates both Gq and G_12/13_ signaling. Phospholipase C (PLC) is activated downstream of Gq, resulting in increased intracellular calcium and PKC activation, both of which are important for dense granule release^19^. We further verified whether TAIII could inhibit the U46619-induced increase in Ca^2+^ concentration upon blockade of the TP receptor. The intracellular Ca^2+^ concentration was measured in fura-2/AM-loaded platelets. As shown in Fig. 5a, TAIII prevented the U46619-induced increase in intracellular Ca^2+^ concentration in a dose-dependent manner. Therefore, we hypothesized that the inhibitory effects of TAIII on TxA2-induced ADP secretion result from the downstream blockade of TP-mediated Gq signaling pathways. To investigate this hypothesis, we first examined whether activation of PKC by PMA could restore dense granule secretion and aggregation in TAIII-treated platelets. As shown in Fig. 5b, exogenous PMA (20 nmol/L), which alone cannot induce ADP secretion and platelet aggregation, completely reversed the inhibitory effect of TAIII on dense granule secretion and platelet aggregation in rat PRP. Accordingly, exogenous *m*-3M3FBS (400 μmol/L), which directly activates PLC, fully restored TAIII-mediated platelet aggregation (Fig. 5d). These results suggest that blocking TP-mediated Gq signaling pathways contributes to the inhibitory effects of TAIII on TxA2-induced ADP secretion and platelet aggregation.

**Figure 6:**
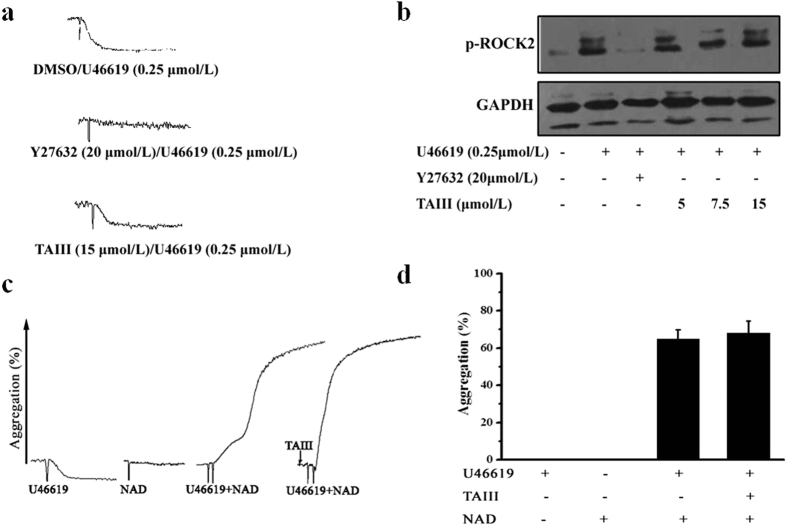
TP-mediated G_12/13_ signaling pathway is unaffected by TAIII. Rat PRP was preincubated with 20 μmol/L Y27632 or 15 μmol/L TAIII for 3 min and then stimulated with 0.25 μmol/L U46619 for 5 min. Platelet shape change and ROCK2 phosphorylation were measured. Typical platelet shape change traces and western blots are shown in **(a)** and **(b)**, and all experiments were performed three independent times. Rat PRP was stimulated with U46619 (0.25 μmol/L), norepinephrine (20 μmol/L) or a combination of both agonists in the presence of 7.5 μmol/L TAIII. Typical platelet aggregation traces are shown in **(c)**. Quantitative evaluation of the data from three independent experiments is shown in **(d)**, and the results are presented as the mean ± SEM.

TP receptor-mediated activation of the Gq and G_12/13_ pathways is dependent on agonist concentration. At lower concentrations, U46619 stimulates the activation of G_12/13_ pathways only, leading to calcium-independent platelet shape change that involves RhoA and p160ROCK (Rho-associated protein kinase) activity in human and mouse platelets^20^. This platelet shape change is blocked by ROCK inhibitors. In our experiment, 0.25 μmol/L U46619 triggered ROCK2 phosphorylation and resulted in platelet shape change without aggregation. Consistent with previous reports, ROCK2 phosphorylation and platelet shape change in response to low-dose U46619 were inhibited by Y-27632, a specific ROCK inhibitor. In contrast, TAIII did not inhibit ROCK2 phosphorylation and platelet shape change induced by 0.25 μmol/L U46619 (Fig. 6a,b). The G_12*/*13_-coupled TXA2 receptor has been reported to synergize with G_z_ signaling pathways to activate platelets^21^,^22^. As shown in Fig. 6c, neither 20 μmol/L norepinephrine nor 0.25 μmol/L U46619 alone induced platelet aggregation, but their combination induced full platelet aggregation. TAIII had no effect on the response to the U46619/adrenaline combination. These results indicate that TAIII does not inhibit TP-mediated G_12/13_ signaling pathways.

### Synergistic Inhibitory Effects of TAIII and Known Antiplatelet Agents on Platelet Aggregation

**Figure 7:**
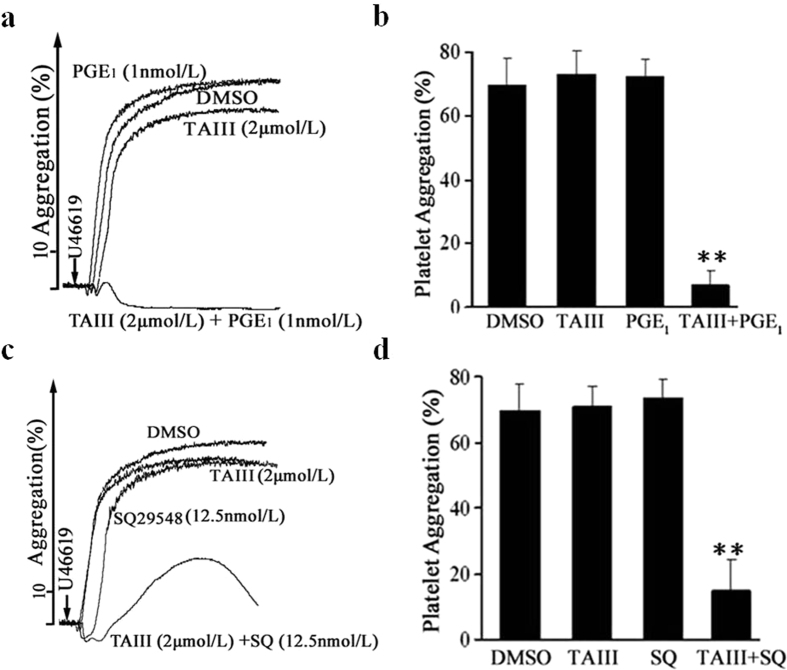
PGE_1_ and SQ29548 enhance the inhibitory effects of TAIII on platelet aggregation. Effects of TAIII and PGE_1_ alone and in combination on U46619-induced aggregation in rat PRP. Rat PRP was incubated with DMSO, TAIII (2 μmol/L), PGE_1_ (1 nmol/L) or a combination of TAIII (2 μmol/L) and PGE_1_ (1 nmol/L) for 3 min, followed by the addition of U46619 (2.5 μmol/L). Platelet aggregation was measured by the turbidimetric method. All experiments were performed three independent times. Typical platelet aggregation traces are shown in **(a)**. Quantitative evaluation of the data from three independent experiments is shown in **(b)**, and the results are presented as the mean ± SEM. Effects of TAIII and SQ29548 alone and in combination on U46619-induced aggregation in rat PRP. Rat PRP was incubated with DMSO, TAIII (2 μmol/L), SQ29548 (12.5 nmol/L) or a combination of TAIII (2 μmol/L) and SQ29548 (12.5 nmol/L) for 3 min, followed by the addition of U46619 (2.5 μmol/L). All experiments were performed three independent times. Typical platelet aggregation traces are shown in **(c)**. Quantitative evaluation of the data from three independent experiments is shown in **(d)**, and the results are presented as the mean ± SEM. Statistical significance was determined by Student’s t-test. **p < 0.01, compared with control platelets.

To examine the characteristics of TAIII in inhibiting platelet aggregation, the synergistic actions of TAIII with known antiplatelet agents were examined. PGE1 is a prostaglandin, which inhibit platelet aggregation though the increase of platelet cAMP levels^17^. As shown in Fig. 7a,b, 1 nmol/L PGE_1_ and 2 μmol/L TAIII exhibited little inhibitory activity against U46619-induced rat platelet aggregation when used alone. Combination treatment with these two agents before U46619 induction strongly inhibited platelet aggregation in rat PRP. SQ 29548 is a highly selective TP receptor antagonist, which inhibits the platelets aggregation induced by U-46619^23^. As shown in Fig. 7c,d, 12.5 nmol/L SQ29548 had no effect on U46619-induced rat platelet aggregation, but the combination of 12.5 nmol/L SQ29548 and 2 μmol/L TAIII nearly completely inhibited U46619-induced platelet aggregation. These results indicate that increasing cAMP concentration with PGE_1_ or blocking TP receptor-mediated signaling pathways with SQ29548 enhances the inhibitory effects of TAIII on platelet aggregation.

### TAIII Modulates Primary Hemostasis and Prevents Thrombus Formation

**Figure 8:**
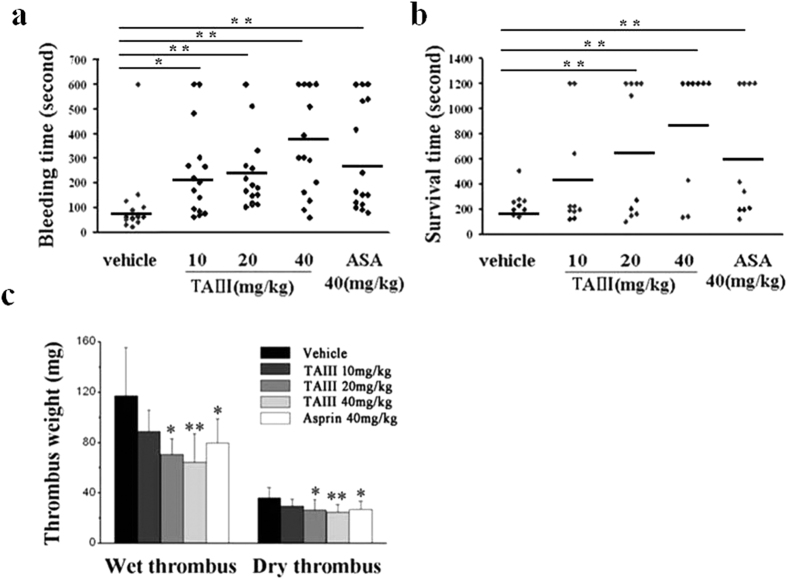
TAIII impairs primary hemostasis and prevents thrombus formation. (**a)** Mice were administered saline (vehicle), TAIII (10, 20 or 40 mg/kg) or aspirin (40 mg/kg) (n = 15 per group), and bleeding time was recorded. **(b)** Mice were administered saline, TAIII (10, 20 or 40 mg/kg) or aspirin (40 mg/kg) (n = 10 per group), and survival time after collagen-epinephrine-induced pulmonary thromboembolism was recorded. The horizontal line indicates the mean bleed time (or survival time); each symbol represents one animal. Statistical significance was determined by the Mann-Whitney test. *p < 0.05, **p < 0.01, compared with the vehicle-treated group. **(c)** Rats were administered saline, TAIII (10, 20 or 40 mg/kg) or aspirin (40 mg/kg) (n = 6 per group), and the wet weight and dry weight of the thrombus were measured. The bar graph represents the mean ± SEM (n = 6). Statistical significance was determined by Student’s t-test. *p < 0.05, compared with the saline-treated group.

To clarify the role of TAIII in platelet function *in vivo*, tail bleeding was observed in mice. As shown in Fig. 8a, the duration of tail bleeding in the vehicle-treated group was 88.9 ± 45.9 s. With increasing doses of TAIII, the tail bleeding time was markedly prolonged to 200.3 ± 162.0, 209.9 ± 148.0, and 396.6 ± 208.9 s; this increase was significant in the group treated with 40 mg/kg/day TAIII compared with the control group (*P* < 0.05). The positive control, aspirin, a well-known antiplatelet agent, also markedly prolonged the tail bleeding time (277.4 ± 188.5 s) at a dose of 40 mg/kg.

To determine the antithrombotic effect of TAIII, mouse pulmonary thromboembolism and rat arterio-venous shunt tests were performed. Intravenous injection of a mixture of collagen and epinephrine into the mouse tail vein caused pulmonary thromboembolism, resulting in 100% death in the control group, with an average survival time of 198.7 ± 54.2 s. Previous oral administration of TAIII resulted in a significant dose-dependent protective effect; the survival time after treatment with 10, 20 or 40 mg/kg TAIII was 427.7 ± 433.17, 677.6 ± 532.54, and 878.2 ± 490.10 s, respectively (Fig. 8b). As a positive control, the survival time of the aspirin (40 mg/kg)-treated group was 625.8 ± 501.9 s.

The insertion of a shunt between the carotid artery and the jugular vein led to the formation of a wet thrombus weighing 117.1 ± 38.3 mg (dry weight 35.9 ± 8.4 mg) in the control group. After administration of 10, 20 or 40 mg/kg TAIII or 40 mg/kg aspirin, the wet weight of the thrombus decreased to 88.6 ± 38.3, 70.3 ± 12.7, 64.3 ± 22.6, and 79.7 ± 19.0 mg, respectively, and the dry weight of the thrombus decreased to 29.5 ± 5.4, 26.6 ± 8.1, 24.4 ± 6.3, and 26.7 ± 6.4 mg, respectively (Fig. 8c). TAIII significantly reduced thrombus formation in a dose-dependent manner. These results indicate that TAIII exhibits strong antithrombotic effects *in vivo* with a low bleeding risk.

## Discussion

The TxA2 pathway is a major contributor to the amplification of initial platelet activation. The effects of TxA2 are mediated by the TP receptor, which is expressed not only in platelets but also in endothelial cells, macrophages, and monocytes^6^,^24^. The TxA2 pathway is therefore a major target in the treatment of cardiovascular disease. Here, we first identified TAIII as a selective inhibitor of TxA2-induced platelet activation. By targeting the Gq-mediated signaling pathway from the TxA2 receptor, TAIII leads to antiplatelet activity *in vitro* and antithrombotic activity *in vivo*.

TAIII, a major active steroidal saponin in *Anemarrhena asphodeloides* Bunge (Liliaceae), possesses a wide spectrum of pharmacological activities, including inhibiting platelet aggregation^12^, tumorigenic behavior^25^,^26^, and inflammation^27^ and improving learning and memory deficits^28^. A turbidimetric assay revealed that TAIII had the strongest inhibitory effect on U46619-induced platelet aggregation among 300 steroidal saponins purified from Chinese medicinal herbs. U46619 is a stable synthetic analog of the endoperoxide prostaglandin H2 (PGH_2_) and acts as a TxA2 (TP) receptor agonist. Using rat platelets as a biologically relevant system, we tested the selectivity of TAIII for the TP receptor versus other G protein-coupled receptors, including thrombin and ADP receptors as well as non-GPCR receptors for collagen, and versus the PKC activator PMA. TAIII dose-dependently inhibited platelet aggregation induced by U46619, AA and low-dose collagen but had little effect on ADP-, thrombin- or PMA-induced aggregation, even at the maximal concentration of 60 μmol/L. Platelet aggregation induced by low-dose collagen or AA is dependent on the release of TxA2, and thus, TAIII was proposed to be a selective inhibitor of TxA2-induced platelet activation. The acute and transient activation of platelet ERK1/2 after stimulation with agonists such as U46619, thrombin and PMA plays a role in promoting platelet aggregation. TAIII significantly suppressed U46619-induced ERK1/2 phosphorylation with little effect on thrombin- and PMA-induced activation of ERK1/2, further confirming that TAIII is a selective inhibitor of TxA2-induced platelet activation. TAIII is a spirostanol saponin consisting of a hydrophilic galactose-glucose disaccharide moiety and a lipophilic aglycone sarsasapogenin. To investigate whether TAIII causes platelet lysis, we measured LDH activity following the addition of TAIII to rat PRP. A 10-min incubation of TAIII with platelets did not significantly increase LDH activity, even at the highest concentration of 60 μmol/L, indicating that TAIII did not affect platelet permeability or induce platelet cytolysis; thus, the activity of TAIII is clearly not related to any detergent role (Supplementary Fig. S1).

TP receptor signal transduction involves Gq and G_12_/G_13_ signaling, which is responsible for platelet activation. Stimulation of Gq family proteins causes activation of PLC-β, resulting in the accumulation of inositol-1,4,5-trisphosphate and diacylglycerol, which in turn activate calcium release from the endoplasmic reticulum and PKC, respectively^29^. Diacylglycerol production has been associated with platelet secretion, and PKC inhibition reduces the release of dense granules from platelets^30^. Stimulation of the G_12_ family proteins G_12_ and G_13_ activates Rho signaling, which modulates several biological reactions. In particular, the TP receptor-mediated platelet shape change is mainly dependent on G_12/13_, whereas aggregation is dependent on Gq^22^. TAIII dose-dependently inhibited U46619-induced rat platelet aggregation, ATP release and calcium mobilization, and restoring the low ADP concentration reversed the inhibitory effects of TAIII, suggesting that the inhibitory effects of TAIII on platelet aggregation are mainly due to the inhibition of ADP secretion from dense granules. PKC activation by PMA or PLC activation by m-3M3FBS restored dense granule secretion and aggregation in TAIII-treated platelets, suggesting that blocking TP receptor-mediated Gq signaling pathways contributes to the inhibitory effects of TAIII on TxA2-induced ADP secretion and platelet aggregation. Signaling through G_12/13_ activates RhoA and hence ROCK, resulting in calcium-independent platelet shape change. However, an inhibitory effect of TAIII on platelet shape change or ROCK activation downstream of G_12/13_ stimulation was not observed. Recent studies have demonstrated that co-stimulation of the G_12/13_ and G_i_ or G_z_ pathways leads to platelet aggregation^21^. In our experiment, co-activation of the G_12/13_ pathways by lower concentrations of U46619 (0.25 μmol/L) and of G_z_ pathways by norepinephrine (20 μmol/L) induced full platelet aggregation that was not inhibited by TAIII. These results indicate that TAIII preferentially suppresses TP receptor-mediated activation of Gq, not G_12/13_, signaling pathways.

Platelet activation involves a complex network of interdependent biochemical processes, through which platelets undergo a sequence of responses: shape change, aggregation, and secretion. Studies using radiolabeled TxA2 analogs as ligands have revealed two classes of binding sites in platelets^29^. These studies also suggested that the two putative binding sites on the receptor may independently mediate shape change and aggregation, as supported by reports that platelet shape change and aggregation can be differentiated by several TxA2 analogs. For example, the TxA2 analog S-145 prevents aggregation and secretion in response to U46619 but itself induces shape change^31^. More interestingly, we observed that TAIII induced shape change while inhibiting U46619-induced rat platelet aggregation and ATP release. Y27632 and SQ29548 both completely inhibited the platelet shape change induced by U46619 but not that induced by TAIII. By contrast, the PLC inhibitor U73122 nearly completely inhibited the TAIII-induced platelet shape change (Supplementary Fig. S4). One model consistent with these observations is that TAIII, by directly or indirectly binding the TP receptor, preferentially suppresses TP receptor-mediated activation of Gq while weakly activating PLC, resulting in platelet shape change.

Efforts to inhibit the production and effects of TxA2 have led to the development of several drugs that target key proteins along the TxA2 pathway, such as the COX and TxS enzymes and the TP receptor. TP antagonists are of particular interest and may have some advantages over aspirin or TxS inhibitors because they block the effects of all TP ligands, including TxA2, isoprostanes, HETEs, and PG endoperoxides. The TP receptor is expressed in numerous cell types related to cardiovascular disease, such as platelets and cells in vascular walls or atherosclerotic plaques^32^. Therefore, TP receptor antagonists may have antiplatelet effects and impact endothelial function and plaque formation. Among the numerous TP antagonists that have been developed, few have reached phase III trials due to toxicity or moderate activity. Ramatroban and seratrodast are both registered in Japan for asthma and allergic rhinitis, which are clearly TxA2-mediated diseases^33^,^34^. TAIII, a major active steroidal saponin in Zhimu, has not been used clinically as a pure compound, but some well-known traditional medicine recipes prepared from Zhimu, such as Er-Mu preparation (EMP) and Zhimu–Baihe herb-pair (ZMBHHP), have been widely used in Oriental countries to treat cough and sputum for thousands of years, and they are effective with limited toxicity. The pharmacological mechanisms of these traditional medicines as cough therapeutics may involve the targeting of TP signaling pathways by TAIII or its analogs.

To develop new therapeutic agents for the treatment of cardiovascular and ischemic disorders, the antiplatelet activity of TAIII was characterized *in vitro* and *in vivo* in the present study. TAIII dose-dependently inhibited U46619-induced rat platelet aggregation and ATP release independent of cAMP elevation and blockade of TxA2 production. Furthermore, increasing cAMP concentration with PGE_1_ or blocking TP-mediated signaling pathways with SQ29548 enhanced the inhibitory effects of TAIII on platelet aggregation. Measuring the duration of tail bleeding is an excellent test for evaluating *in vivo* platelet function. We observed that the tail bleeding time was significantly prolonged in mice treated with TAIII due to its inhibitory effects on platelet function. The *ex vivo* anti-platelet properties of TAIII were further verified in an animal model of thrombosis. TAIII potently protected mice against death due to pulmonary thrombosis in a dose-dependent manner. Evaluation of the antithrombotic properties of TAIII using an arterio-venous shunt in rats revealed that oral TAIII significantly reduced the weight of the formed thrombus in a comparable manner to aspirin, a conventional antithrombotic agent. TAIII, by targeting the Gq-mediated signaling pathway of the TP receptor, exhibited stronger antithrombotic effects *in vivo* with lower risks of bleeding compared with aspirin. TAIII may therefore be an excellent candidate for the treatment of thrombotic disorders or thromboxane-mediated diseases.

In conclusion, our results demonstrate that TAIII is a potent and selective inhibitor of TxA2-induced platelet aggregation that abolishes ADP secretion independent of increases in cAMP or cGMP and blockade of TxA2 production. Furthermore, TAIII preferentially suppresses the thromboxane receptor-mediated activation of Gq, not G_12/13_, signaling pathways, and increasing cAMP concentration or blocking TP-mediated signaling enhances the inhibitory effects of TAIII on platelet aggregation. Characterizing the mechanism of the antiplatelet activity of TAIII and evaluating its antithrombotic activity *in vivo* may facilitate the development of TAIII as a novel antithrombotic agent.

## Materials and Methods

### Reagents

U46619, AA, thrombin, SQ29548, Y27632, U73122, SNAP, the cGMP EIA kit and the TxB2 EIA kit were purchased from Cayman Chemical (Ann Arbor, MI, USA). ADP, heparin, PMA, fura 2-AM, bovine serum albumin (BSA) and aspirin were purchased from Sigma (St. Louis, MO, USA). 2,4,6-Trimethyl-*N*-[3-(trifluoromethyl)phenyl] benzenesulfonamide (*m*-3M3FBS) was purchased from Calbiochem (Darmstadt, Germany). Antibodies against p44/42 MAPK (ERK1/2), phospho-p44/42 MAPK (pERK1/2) (Thr^202^/Tyr^204^), phospho-ROCK2 (Ser^1366^), and GAPDH, as well as the enhanced chemiluminescence (ECL) system detection kit and HRP-conjugated anti-mouse IgG, were obtained from Cell Signaling Technology (Beverly, MA, USA). The BCA protein assay kit was purchased from Thermo Scientific (Waltham, MA, USA). CHRONO-LUME reagent and collagen were purchased from Chrono-Log Corp (Havertown, PA, USA). The LANCE^TM^ cAMP kit was purchased from Perkin Elmer (Waltham, MA, USA). Sepharose CL-2B was purchased from GE Healthcare (Uppsala, Sweden). Compound purification was described in a previous study^35^. Compounds were dissolved in dimethyl sulfoxide (DMSO). All other chemicals were of reagent grade.

### Animals

Male Wistar rats (8 weeks of age, 200–250 g) and male Balb/c mice (7 weeks of age, 18–22 g) were purchased from the Laboratory Animal Center, Chinese Academy of Medical Sciences (Beijing, China), and were allowed to acclimate to a specific pathogen-free (SPF) animal facility with free access to water and feed. Animal welfare and experimental procedures were performed in accordance with the National Institutes of Health Guidelines for the Care and Use of Laboratory Animals. This study was approved by Beijing Experimental Animal Ethics Committee established by the Beijing People’s Government. All surviving animals were euthanized by cervical dislocation and discarded.

### Preparation of Platelet-rich Plasma (PRP) and Washed Platelets

Wistar rats were anesthetized using sodium pentobarbital (40 mg/kg i.p.), and blood was obtained by cardiac puncture and mixed with one-ninth volume of 200 IU/ml heparin. PRP was prepared by centrifugation of anticoagulant-treated blood at 2300× *g* for 1 min at room temperature (RT), and platelet-poor plasma (PPP) was prepared by subsequent centrifugation at 3000× *g* for 15 min. Washed platelets were obtained as follows. PRP from rat blood anti-coagulated with acid citrate dextrose solution (ACD, 85 mmol/L sodium citrate, 71 mmol/L citric acid and 110 mmol/L glucose) was obtained by centrifugation at 2300× *g* for 1 min at RT. Platelets were separated from plasma proteins by gel filtration on Sepharose CL-2B and suspended in Tyrode’s buffer (5.5 mmol/L glucose, 2 mmol/L KCl, 12 mmol/L NaHCO_3_, 137 mmol/L NaCl, 0.3 mmol/L NaPHO_4_, and 1 mmol/L MgCl_2_, pH 7.4) containing 3.5 mg/ml BSA. After gel filtration, the platelet suspension was adjusted to a final concentration of 300× 10^9^/L in Tyrode’s buffer.

### Platelet Aggregation and Secretion

Platelets were incubated at 37 °C, and aggregation was measured using a turbidimetric method in a dual-channel Lumi-aggregometer (Chronolog, Havertown, PA, USA) under continuous stirring. Platelet suspensions incubated with various concentrations of compounds or DMSO for 3 min were stimulated with various agonists. To eliminate the effect of vehicle on aggregation, the final concentration of DMSO was fixed at 0.5% (vol/vol). The extent of inhibition of platelet aggregation was expressed as percent inhibition (*X*) using the following equation: *X* (percent) = (1−*B*/*A*) × 100%, where *A* is the maximum aggregation rate of vehicle-treated platelets, and *B* is the maximum aggregation of compound-treated platelets. ATP secretion was measured by incubating 238 μl of the platelet suspension with 12 μl of a luciferin/luciferase reagent at 37 °C under continuous stirring in the Lumi-aggregometer. After 3 min of incubation with TAIII, agonists were added to the suspension, and tracings of aggregation and ATP secretion were recorded for at least 5 min. Platelet shape change was estimated by measuring the maximum curve height below baseline.

### Measurement of cAMP Levels

PRP (500 μl) was incubated with TAIII or PGE_1_ at 37 °C for 3 min, and the platelets were then precipitated by centrifugation in the presence of 10 mmol/L EDTA. The supernatant was discarded, and the platelets were resuspended in 500 μl of Hank’s balanced salt solution. The platelet suspension was heated for 5 min at 100 °C, and the supernatant was collected after centrifugation. The cAMP level in the supernatant was measured in triplicate using the LANCE^TM^ cAMP Kit as described by the manufacturer.

### Measurement of cGMP Levels

PRP (500 μl) was incubated with TAIII or SNAP at 37 °C for 3 min, and the reactions were stopped by the addition of an equal volume of ice-cold 12% (wt/vol) trichloroacetic acid. The samples were mixed and centrifuged at 2000× g for 5 min at 4 °C. The supernatants were removed, and the pellets were washed 4 times with 5 volumes of water-saturated diethyl ether and then lyophilized. The cGMP concentration was measured using a cGMP EIA kit.

### Measurement of TxA2 Production

After incubating PRP with TAIII or aspirin at 37 °C for 3 min, the platelets were stimulated with AA for 5 min and then removed by centrifugation in the presence of 2 mmol/L EDTA. The plasma samples were diluted 1:50 with PPP, and thromboxane B2, the stable metabolite of TxA2, was measured using a thromboxane B2 EIA kit according to the manufacturer’s protocol.

### Measurement of Intracellular Calcium Concentration

Rat PRP was centrifuged at 800× g for 10 min, and the supernatant was removed. The platelets were resuspended in Tyrode’s buffer without calcium, and the platelet density was adjusted to 750 × 10^9^/L. Platelets were loaded with 10 μmol/L fura-2/AM for 45 min at 37 °C in the dark. Excessive dye was removed by centrifugation. The pelleted platelets were then resuspended in PPP and diluted to an approximate density of 300 × 10^9^/L. Calcium chloride was added at a final concentration of 1.25 mmol/L. The platelet suspension was pre-incubated with TAIII at 37 °C for 3 min, and calcium mobilization in response to U46619 was measured based on A340 and A380 using a spectrofluorometer (Varian, Inc., Walnut Creek, CA).

### Western Blot Analysis

PRP was prepared for western blot analysis by the addition of 1/10 volume of 1.0 mol/L formaldehyde in 154 mmol/L NaCl. The samples were placed on ice for 30 min, followed by centrifugation at 10,000 × *g* for 1 min at 4 °C. The supernatant was completely removed, and the pellet was resuspended in lysis buffer (100 mmol/L Tris-HCl, pH 6.8, 4% (m/v) SDS, 20% (v/v) glycerol, 200 mmol/L β-mercaptoethanol, 1 mmol/L PMSF, 0.1 mmol/L NaF and DTT). The samples were heated for 10 min at 100 °C, and total protein was extracted. The protein concentration in the supernatant was detected by BCA protein assay. Equal amounts of protein were separated by *sodium dodecyl sulfate-*polyacrylamide gel electrophoresis (SDS-PAGE) and transferred to nitrocellulose membranes. The membranes were probed with specific primary antibodies and an HRP-conjugated secondary antibody. Bound antibodies were detected with an ECL detection kit.

### Tail Bleeding Time Experiment

Balb/c mice were randomly divided into five groups and orally administered 100 μl of TAIII twice daily for 5 days at doses of 10, 20 or 40 mg/kg body weight or 40 mg/kg body weight of aspirin as a positive control. The same volume of saline was administered as a vehicle. One hour after the last administration, the mice were anesthetized with sodium pentobarbital (30 mg/kg i.p.). The tails were transected 2 mm from the tip and vertically immersed in saline at 37 °C. The time until continuous blood flow ceased for >30 s was measured, with a maximum observation time of 600 s (longer bleeding times were assigned a value of 600 s).

### *In Vivo* Antithrombotic Effect Assay

The mouse pulmonary thromboembolism test was performed as previously described^36^. In brief, Balb/c mice were randomly allocated to five groups and orally administered 100 μl of TAIII twice daily for 5 days at doses of 10, 20, or 40 mg/kg body weight or 40 mg/kg body weight of aspirin as a positive control. The same volume of saline solution was administered as a vehicle. One hour after the last administration, 100 μl of a collagen solution (114 μg/mouse) and epinephrine (13.2 μg/mouse) was injected into the tail vein to induce pulmonary thrombosis. The number of dead or paralyzed mice was recorded for up to 20 min.

The oral anti-thrombotic activity of TAIII was determined in a rat arteriovenous-shunt model. In brief, male Wistar rats were orally administered 10 ml/kg of TAIII (10, 20 or 40 mg/kg body weight) or aspirin (40 mg/kg body weight) twice daily for 5 days. The same volume of saline was administered as a vehicle. One hour after administration, the rats were anesthetized with sodium pentobarbital (40 mg/kg i.p.). Two 12-cm polyethylene tubes (0.6 and 0.9 mm inner and outer diameters, respectively) linked to a central tube (10 cm long, 0.9 cm inner diameter) containing a 7-cm silk thread and filled with a heparin saline solution (25 IU/ml) were placed between the right carotid artery and the left jugular vein. The central part of the shunt was removed after 15 min of blood circulation, and the silk thread carrying the thrombus was pulled out. The weight of the thrombus was determined.

### Statistical Analysis

Results are presented as the mean ± SEM. Analysis of variance (ANOVA) was performed by two-tailed Student’s t-test. The Mann-Whitney test was used to analyze bleeding time and survival time. *P* values less than 0.05 were considered statistically significant.

## Additional Information

**How to cite this article**: Cong, Y. *et al*. Timosaponin AIII induces antiplatelet and antithrombotic activity via Gq-mediated signaling by the thromboxane A2 receptor. *Sci. Rep.* **6**, 38757; doi: 10.1038/srep38757 (2016).

**Publisher's note:** Springer Nature remains neutral with regard to jurisdictional claims in published maps and institutional affiliations.

## Supplementary Material

Supplemental Material
