# Supplementary material for: Timosaponin AIII induces antiplatelet and antithrombotic activity via Gq-mediated signaling by the thromboxane A2 receptor
Source: Sci Rep. 2016 Dec 9;6:38757. doi: 10.1038/srep38757 (PMC5146924; doi:10.1038/srep38757)
Supplement: Supplemental Material [file srep38757-s1.doc]

**Timosaponin AIII induces antiplatelet and antithrombotic activity via Gq-mediated signaling by the thromboxane A2 receptor**

Yue Cong ^1,2^, Limei Wang ^1^, Renjun Peng ^1^, Yang Zhao ^3^, Fan Bai ^1^, Chao Yang^1^, Xiaolan Liu ^1^, Daqian Wang ^2^, Baiping Ma ^3^, and Yuwen Cong ^1 *^

**SUPPLEMENTAL MATERIAL**

**Materials and methods**

**Preparation of Human Washed Platelets and Platelet aggregation assay**

Venous blood was collected from healthy volunteers in to the tube with 3.2% trisodium citrate. Blood was centrifuged at 200g for 10 min at RT to obtain PRP. Platelets were separated from plasma proteins by gel filtering on Sepharose CL-2B using Tyrode’s buffer. Platelet aggregation was measured using a turbidimetric method in a dual-channel Lumi-aggregometer under continuous stirring. This study has been approved by Ethics Committee of Beijing Institute of Radiation Medicine in accordance with Helsinki declaration for the Use of Human Subjects. We obtained informed consent from all donors.

**Measurement of LDH leakage**

PRP (500 μl) was incubated with TAIII or 1% Triton X 100 (positive control) at 37°C for 10 min, and then the platelets were precipitated by centrifugation at11000 ***g*** for 3 min. Plasma LDH was measured by kit method, the assay was performed in a time course of decrease in NADH absorbance at 340 nm for 1 min.

**Results**

**
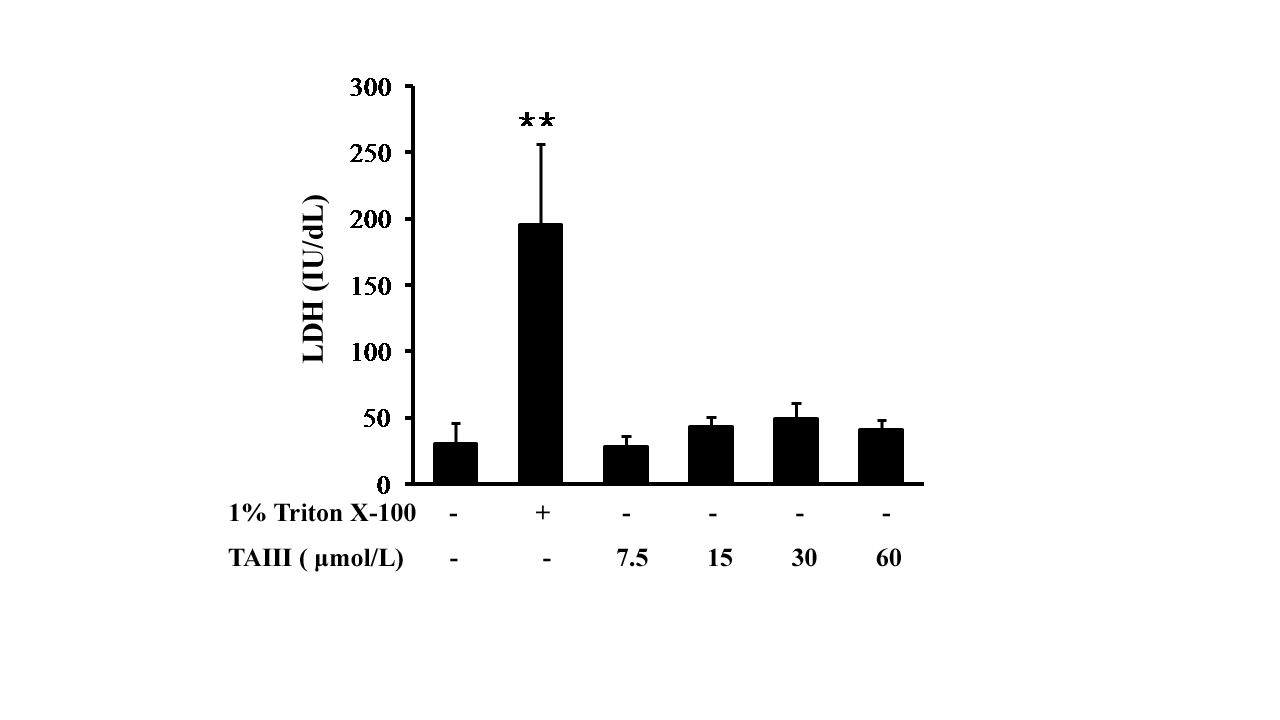
**

**Figure S1.** Effect of TAIII on LDH release in platelets. Rat PRP were treated with increasing concentrations of TAIII (0–60 μmol/L) or 1% TritonX 100 for 10 min at 37℃ and LDH was measured. Results are means ± SEM (n = 4), Statistical significance was determined by Student’s t-test, ***p* < 0.01, compared with the control platelets.

**A**

**
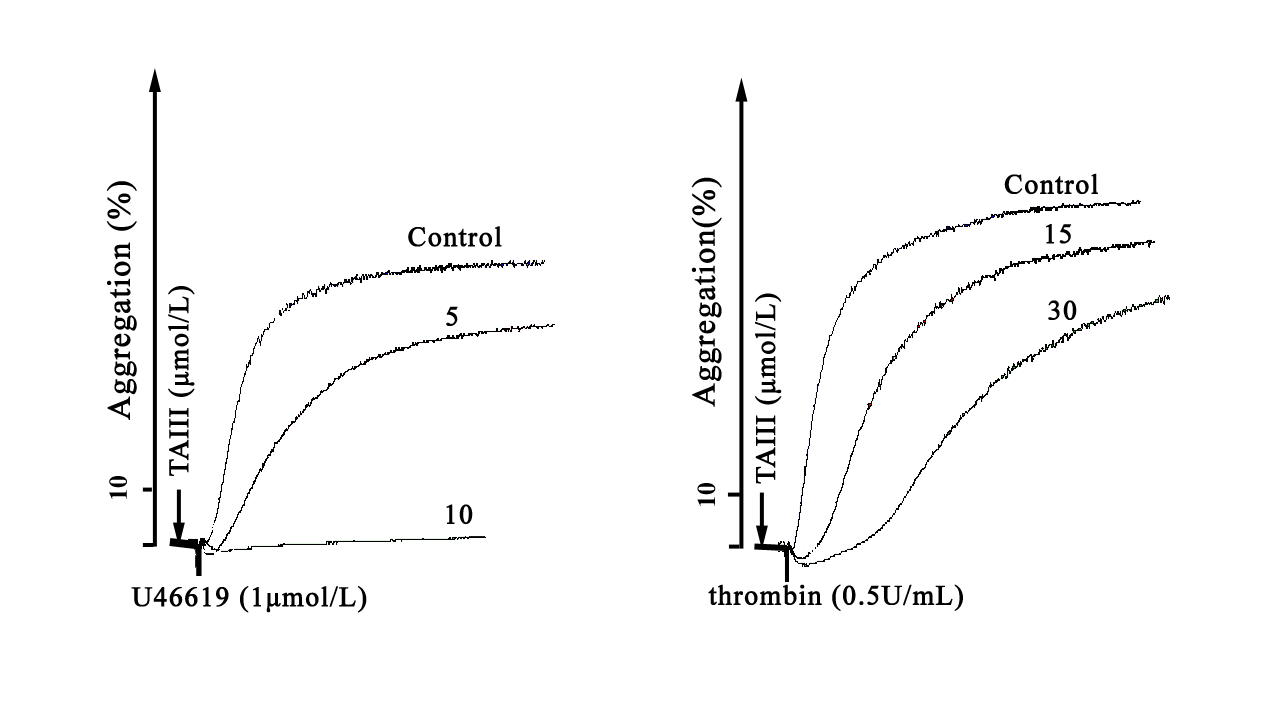
**

**B**

**
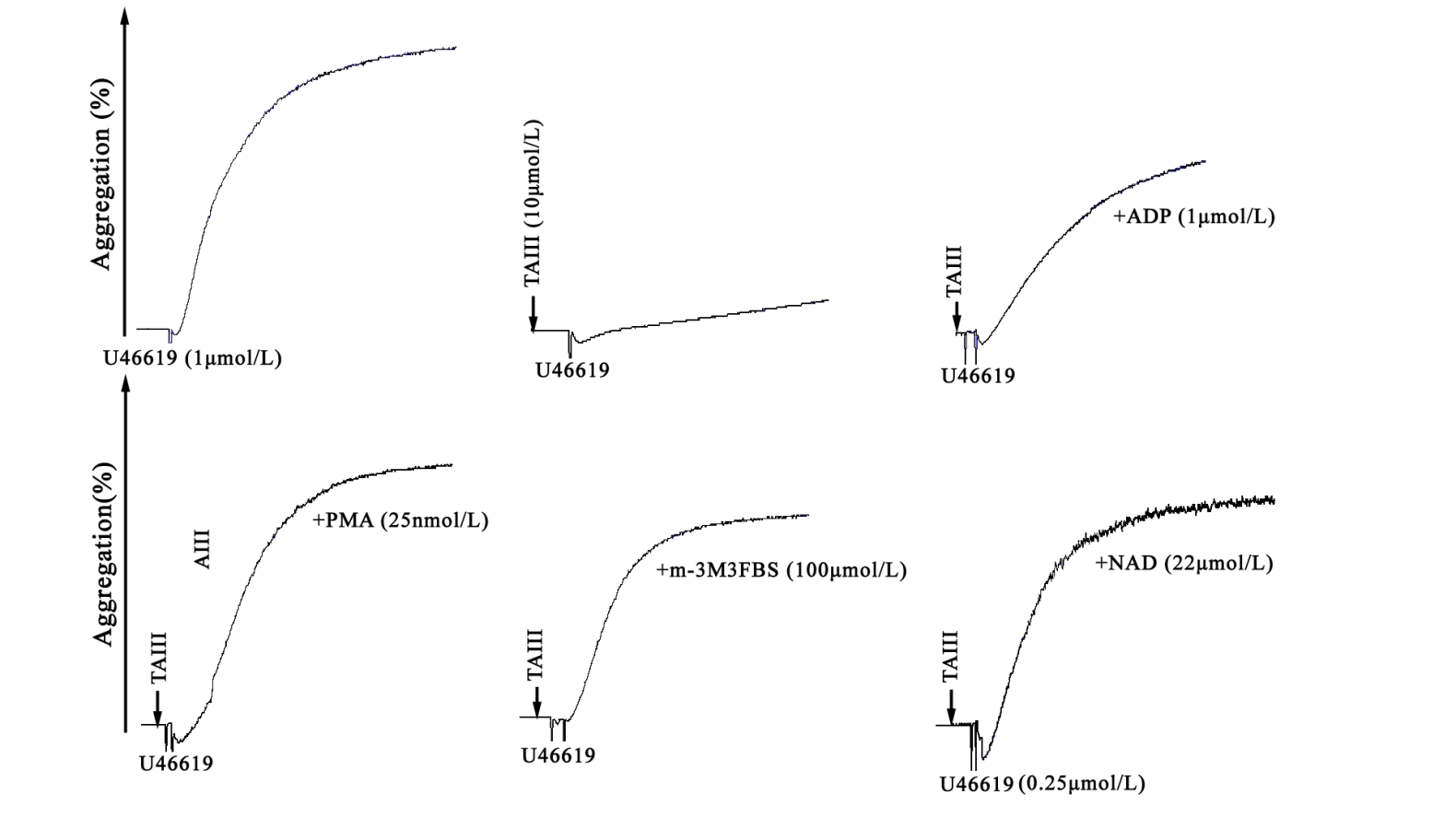
**

**Figure S2.TAIII inhibits human platelets aggregation via the Gq-mediated signaling pathway of the TP receptor. A,** Huamn washed platelts was preincubated with increasing concentrations of TAIII (0–30 μmol/L) for 3 min and then stimulated with U46619 (2.5 μmol/L) or thrombin (0.5 IU/ml). B, Exogenous ADP (1μmol/L), PMA (25nmol/L) or *m*-3M3FBS (100 μmol/L) restored TAIII-inhibited platelet aggregation and dense granule secretion induced by U46619 (1μmol/L); TAIII was not able to inhibit platelet aggregation induced by a combination of U46619 (0.25μmol/L) and NAD (22μmol/L). Typical platelet aggregation traces are representative of three independent experiments.


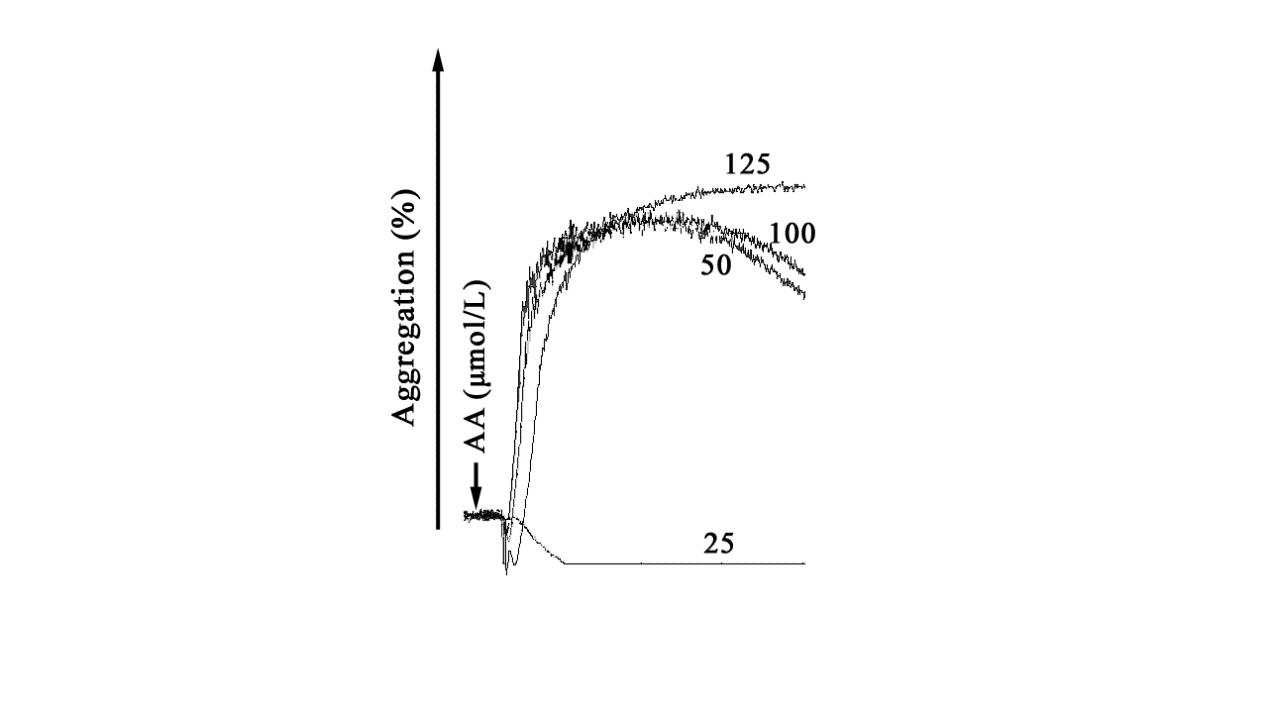


**Figure S3. AA induces platelet aggregation in rat PRP.** Rat PRP was stimulated with increasing concentrations AA (25-125μmol/L). Typical platelet aggregation traces are shown.


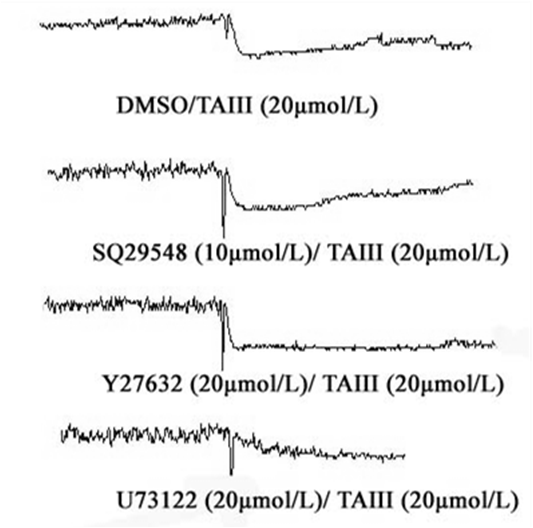


**Figure S4. TAIII induced-platelet shape change is inhibited by the PLC inhibitor U73122.** Rat PRP was stimulated with 20μmol/L TAIII or pretreated with ROCK inhibitor Y27632, TP antagonist SQ29548 or PLC inhibitor U73122 before TAIII. Platelet shape change was measured by turbidimetric method. Typical platelet shape change traces are shown, and all experiments were done in three independent experiments.
